# Endemic characteristics of infantile visceral leishmaniasis in the People’s Republic of China

**DOI:** 10.1186/1756-3305-6-143

**Published:** 2013-05-17

**Authors:** Qing Fu, Shi-Zhu Li, Wei-Ping Wu, Yan-Yan Hou, Song Zhang, Yu Feng, Li-Ping Zhang, Lin-Hua Tang

**Affiliations:** 1 National Institute of Parasitic Diseases, Chinese Center for Disease Control and Prevention, Shanghai 200025, People’s Republic of China; 2 Key Laboratory of Parasite and Vector Biology, Ministry of Health, Shanghai 200025, People’s Republic of China; 3 WHO Collaborating Center for Malaria, Schistosomiasis and Filariasis, Shanghai 200025, People’s Republic of China; 4 Center for Disease Control and Prevention of Xinjiang Uygur Autonomous Region, Urumqi 830002, People’s Republic of China; 5 Center for Disease Control and Prevention of Gansu Province, Lanzhou 730020, People’s Republic of China; 6 Center for Disease Control and Prevention of Sichuan Province, Chengdu 610041, People’s Republic of China

**Keywords:** Endemic, Infantile, Visceral leishmaniasis, China

## Abstract

**Background:**

Visceral leishmaniasis (VL) was once a severe parasitic disease in China. Thanks to the great efforts of integrated control, VL was eliminated in most epidemic areas, except for certain western provinces (autonomous region) at the end of 1950s. From then on, VL gained less attention and has seemed to spread, especially in the last 15 years. Infants are the most important population threatened by VL. However, there have been few studies on the endemic characteristics of infantile VL in China.

**Methods:**

Infantile VL cases were collected from the online National Infectious Diseases Reporting System (NIDRS). Statistical description and inference was used to reveal the endemic characteristics in gender, age group, time and regionalism. Spatial analysis was carried out to explore the high risk area for infantile VL in China.

**Results:**

A total of 1093 infantile VL cases were reported from 2006 to 2012. There was no statistically significant difference in gender over time. The minimum, maximum and mean age of these cases was 1.1, 35.9 and 13.8 months, respectively. Among them 86.92% were under 2 years of age, and there was a statistically significant difference among age groups over time. An incidence peak appeared in 2008-2009, most cases were distributed in the months September to December, and there was a tail-raising effect in the coming two months of the next year. More than 98% of cases were reported in Xinjiang Uygur Autonomous Region, Gansu Province and Sichuan Province, accounting for 61.02%, 32.75% and 4.57%, respectively. A total of 56 counties reported infantile VL cases, with the cumulative incidence ranging from 0.02 to 24.57%. There were two main zones of high endemicity for infantile VL in China. The monthly incidence clearly coincides with the number of towns where infantile VL cases were reported. Three fatalities were reported during the study period, the case fatality rate was 2.75‰.

**Conclusions:**

The endemic situation of infantile VL is serious, and there are several active foci of infantile VL prevalence in China. VL has emerged as a severe threat to infants of endemic regions in China.

## Background

Visceral leishmaniasis (VL) is a potentially fatal parasitic disease, which is caused by several species of intracellular protozoans belonging to the genus *Leishmania*[1-3]. Infected individuals may develop the most severe clinical syndrome, characterized by prolonged fever, splenomegaly, weight loss, hepatomegaly and pancytopenia, with more than 90% case-fatality in the absence of treatment [4]. VL is endemic in more than 62 countries, with an estimated 500 000 new patients each year worldwide [5,6]. In the past, VL severely prevailed in 16 of 34 provinces (municipalities or autonomous regions) of the People’s Republic of China [7]. After the implementation of the comprehensive control campaign, VL had been eliminated in most eastern provinces of China [8,9]. However, in western provinces (autonomous regions), VL is still a serious health problem, the protozoan parasites are *Leishmania donovani* and *Leishmania infantum*, transmitted by the sandfly *Phlebotomus longiductus*, *Phlebotomus wui* and *Phlebotomus chinensis*[10,11]. Infants are the important population threatened by VL in China, new cases ceaselessly occur each year, which should be taken seriously. The objective of this article is to evaluate the endemic status on infantile VL in China, as well as to provide control suggestions.

## Methods

### Definition of infantile VL

A child who is infected by VL from the time of birth to approximately 3 years of age.

### Source of data

Data were collected from the online National Infectious Diseases Reporting System (NIDRS) from January 1st 2006 to December 31st 2012, which covered all notifiable diseases, including VL. Each record included a unique reference number, name, gender, date of birth and diagnosis, home address, etc. The inaccurate and repeated case reporting records were excluded, only the final-judgment cases were saved. Relevant population data was collected from the departments of immunization of provincial Centers for Disease Control and Prevention (CDC) and the national census database from the Bureau of Statistics. The electronic boundaries of counties were retrieved from the national fundamental geographic information system.

### Quality control

Provincial CDCs were responsible for checking and revising the local case reporting records. The National Institute of Parasitic Diseases will check and verify each case reported from the non-endemic areas. Furthermore, an assessment in 4 counties, namely Kashi City, Shufu County, Shule County and Jiashi County was conducted to evaluate the data quality in terms of the precision, reliability, completeness and concordance.

### Data analysis

Infantile VL cases were grouped by gender, age group, time and regionalism, respectively, the Mathsoft Axum 7.0 and SPSS12.0 was used for epidemiological analysis. The cumulative incidence of infantile VL of each county was calculated and analyzed in ArcGIS 9.3 for spatial analysis of infantile VL. The level of statistical significance was set at *P* = 0.05.

### Ethics statement

The study protocol was approved by the Ethics Review Committee of the National Institute of Parasitic Disease of China CDC. Data were collected from the online NIDRS, we did not carry out any field investigation, detection and experiments on patients or their sample. Therefore, there were no ethical issues.

## Results

### Data quality

From January 1st 2010 to June 30th 2012, 196 VL paper reporting cards from 4 counties were compared with those of NIDRS. The underreporting rate was 2.55% (5/196), the repeat rate was 14.80% (29/196), completion rate was 83.67% (164/196), and the concordance rate of key items in NIDRS compared with those on the cards was 97.96% (192/196).

### Endemic characteristics

#### Gender distribution

During the period from January 1st 2006 to December 31st 2012, 1093 infantile VL cases were reported in NIDRS (Table 1). As shown, 59.56% (651/1093) were male, and the sex ratio was 147:100. There was no statistically significant difference in gender over time (*χ*^2^ = 8.60, *P* > 0.05).

**Table 1:** Gender and age distribution of infantile VL cases in China

**Year**	**Gender**		Age* group		
	**Male**	**Female**	**(0-1) y**	**[1-2) y**	**[2-3) y**
2006	46	17	27	30	6
2007	69	48	56	54	7
2008	168	128	179	94	23
2009	162	120	138	106	38
2010	98	68	70	70	26
2011	62	30	18	50	24
2012	46	31	19	39	19
Total	651	442	507	443	143

* Age = date of diagnosis - date of birth.

#### Age distribution

Infants were divided into three age groups, from birth to 1 year (not including 1 year), 1 to 2 years (not including 2 years) and 2 to 3 years (not including 3 years). During the study period, the number of cases of the three age groups was 507, 443 and 143, respectively. The minimum, maximum and mean age of the total cases was 1.1 months, 35.9 months and 13.8 months, respectively. As shown 86.92% (950/1093) of cases were infants under 2 years of age (Table 1). There was a statistically significant difference among age groups over time (*χ*^2^ = 82.50, *P* < 0.05), the number of VL cases in the 2 to 3 year age group was significantly less than that of the other two groups.

#### Time distribution

**Figure 1:**
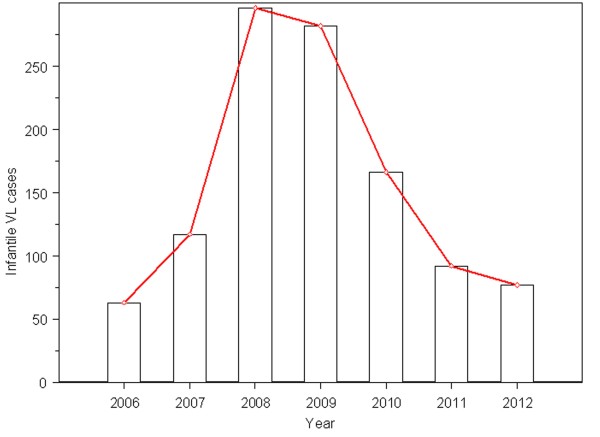
The yearly number of infantile VL cases in China from 2006 to 2012.

**Figure 2:**
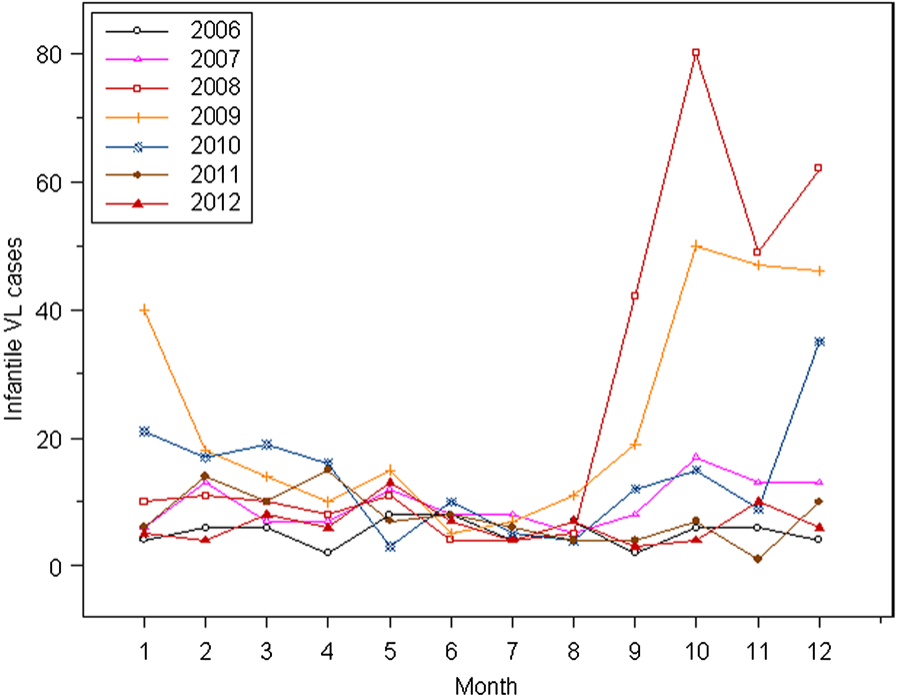
The monthly number of infantile VL cases in China from 2006 to 2012.

The annual average of 156 infantile VL cases was reported during the study period. It showed that there was an incidence peak in 2008-2009 (Figure 1), accounting for 52.88% (578/1093). Cases were grouped by month, 53.07% (580/1093) distributed in the months September to December, which were the peak time of incidence of infantile VL. The monthly number of infantile VL cases indicated a strongly seasonal distribution pattern, with a peak in autumn according to the Chinese lunar calendar. Moreover, there was a tail-raising effect in the coming two months of the next year (Figure 2).

#### Geographical distribution

A total of 8 provinces (autonomous regions) reported infantile VL cases (Table 2). Most of the cases were distributed in Xinjiang Uygur Autonomous Region, Gansu Province and Sichuan Province, which accounted for 61.02% (667/1093), 32.75% (358/1093) and 4.57% (50/1093), respectively. Sporadic infantile VL cases were reported in Inner Mongolia Autonomous Region, Shanxi Province and Shaanxi Province. Furthermore, 3 infantile VL cases were reported as imported cases, two of which were in Zhejiang Province, and the other one was in Hunan Province.

**Table 2:** Geographical distribution of infantile VL cases in China

**Province (Autonomous Region)**	**Year**							**Total**
	**2006**	**2007**	**2008**	**2009**	**2010**	**2011**	**2012**	
Xinjiang	23	40	249	205	105	18	27	667
Gansu	33	64	39	67	55	60	40	358
Sichuan	4	11	8	8	6	8	5	50
Inner Mongolia	1	1	0	1	0	0	0	3
Shanxi	1	0	0	1	0	3	2	7
Shaanxi	0	1	0	0	0	1	3	5
Zhejiang*	1	0	0	0	0	1	0	2
Hunan*	0	0	0	0	0	1	0	1

* Cases were confirmed as imported cases from other endemic province.

**Figure 3:**
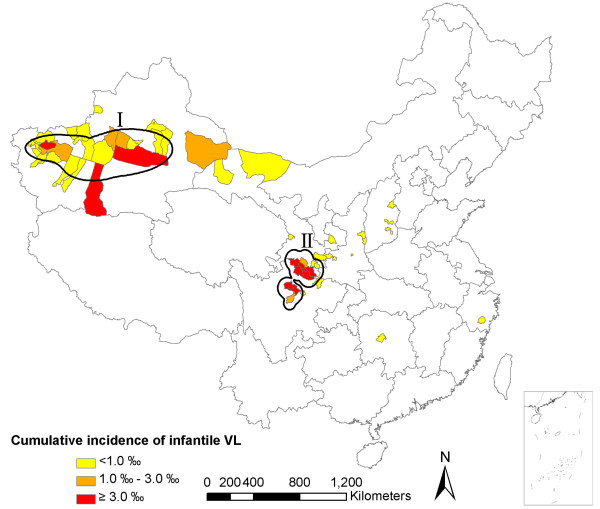
Choropleth map of cumulative incidence of infantile VL in China from 2006-2012.

56 counties (cities) reported infantile VL cases during the study period. Grouped by county, the cumulative incidence of infantile VL of each county was calculated and joined to the map layer in ArcGIS 9.3, with county name matching. Cumulative incidences were divided into 3 quantities with gradual colors (Figure 3). Eleven counties with red color indicated the cumulative incidence was higher than 3.00‰, three of which were over 10.00‰, they are Wen County of Gansu Province (24.57‰), Jiashi County of Xinjiang Uygur Autonomous Region (17.31‰) and Zhouqu County of Gansu Province (11.04‰). In addition, Wudu City and Diebu County of Gansu Province, Yuli County of Xinjiang Uygur Autonomous Region, and Heishui County of Sichuan Province, their cumulative incidence was all higher than 6.00‰. Cumulative incidences were analyzed in Spatial Analyst extension, and contours were created on the basis of density analysis, As a result, there were two main zones (IandII) of high endemicity for infantile VL in China from 2006 to 2012.

**Figure 4:**
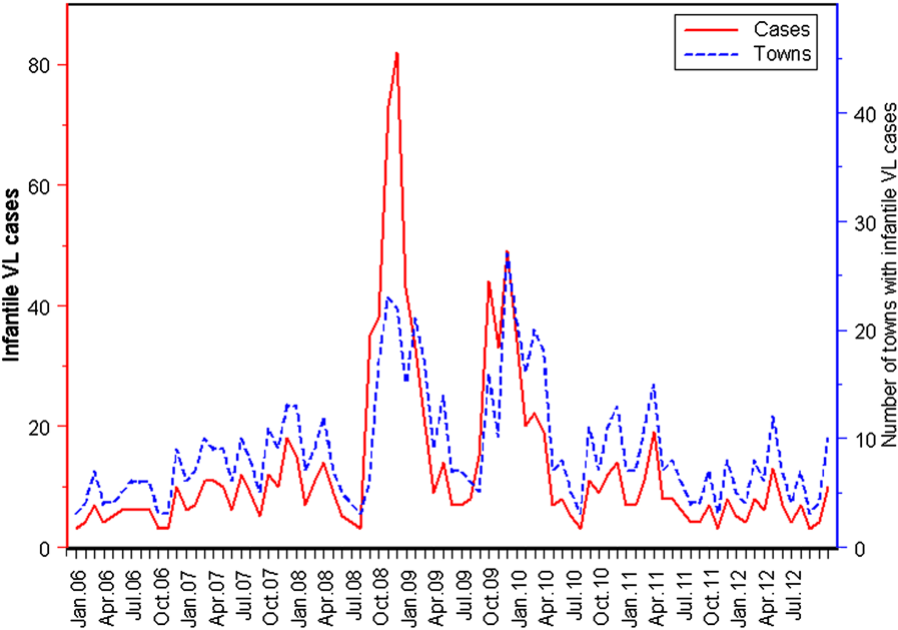
The monthly number of infantile VL cases and towns with cases from 2006 to 2012.

The number of infantile VL cases and the number of towns with infantile VL cases of each year were grouped by month, the relationship between the monthly number of cases and towns is presented in Figure 4. It showed that there was a large peak of infantile VL incidence that occurred in December 2008 (82 cases), and the monthly number of cases clearly coincides with the number of towns where infantile VL cases were reported.

#### Deaths

Of these 1093 well-documented infantile VL cases, three fatalities were reported in 2007 (Table 3), the case fatality rate was 2.75‰.

**Table 3:** Deaths of infantile VL in China from 2006-2012

**Province (Autonomous Region)**	**County**	**Gender**	**Year of death**	**Age at death**
Sichuan	Heishui	female	2007	12 months
Xinjiang	Kuerle	female	2007	10 months
Xinjiang	Urumqi	male	2007	13 months

## Discussion

VL causes 500 000 cases each year with more than 90% in India, Bangladesh, Brazil, Nepal and Sudan [4]. At present, a VL elimination program was launched by The WHO South-East Asia Region in 2005 [12,13], which indicates that the global elimination of VL is put on the agenda. However, in China and some other endemic countries, VL has been neglected and seems to have spread in recent years [14-17]. In the early 1950s, there were approximately 530 000 VL cases distributed in more than 660 counties of 16 provinces in China [7]. At that time, governments at different levels paid a great deal of attention to VL control to initiate the national control program. VL in China can be classified into three types by different topographic features, *Leishmania* species, vector species and reservoir hosts [18,19], namely anthroponotic type VL (AVL), mountainous sub-type of zoonotic VL (MST-ZVL) and desert sub-type of zoonotic VL (DST-ZVL) [20]. In AVL endemic areas, the control strategy placed emphasis on mass screening and treatment of patients, combined with vector control using indoor or outdoor spraying of insecticides [10]. A large number of patients were found to be infected and were treated using sodium stibogluconate [21], meanwhile the density and natural infection rate of sandflies gradually decreased [22], as a result, prevalence of AVL was effectively controlled by the end of the 1950s in China. On the contrary, because of the exophilic behaviour of the sandfly in MST-ZVL and DST-ZVL endemic areas, there was little effect on vector control by spraying of insecticides [23,24]. In MST-ZVL endemic areas, we adopted the strategy with emphasis on extinction of infected dogs and prohibition of raising domestic dogs, combined with deltamethrin bathing for dogs, which markedly interrupted the transmission of MST-ZVL [25,26]. Although there were no available means to control DST-ZVL, because of the unknown source of infection and the wild nature of the vector, the number of patients reported was low and they could easily recover through treatment in hospitals. The various control strategies for different types of VL had made great contributions to elimination of VL in China by the end of 1950s, which will be of referential significance to current VL control. Following the mass prevention and control, VL was basically eliminated in China. Since then, because of the decreasing incidence, VL control has been ruled out from the priority agenda. Due to lack of a sound surveillance system and resources for control activities, VL is now one of the neglected tropical diseases in China. According to the data collected from 1990 to 1999, VL was prevalent or sporadically endemic in 43 counties of 6 western provinces (autonomous regions), which were Xinjiang, Gansu, Sichuan, Shaanxi, Shanxi and Inner Mongolia [10]. More importantly, re-emergence and outbreaks of VL occur intermittently in China [27-29]. In recent years, two outbreaks of VL took place in west Xinjiang Uygur Autonomous Region, namely Shache County of Kashgar Region in 2006 and Jiashi County of Kashgar Region in 2008 to 2009.

It is feasible to evaluate the normative of VL cases reporting in China, as VL is a notifiable disease by law. VL cases have to be reported by CDC and hospitals at different levels since 2006, so the completeness and reality can be largely ensured. The results of assessment showed a low underreporting rate, a high completeness and concordance rate, so the quality of available data from NIDRS is relatively ideal for analysis on endemic characteristics. Nevertheless, VL is often a hidden health problem, which can unfold without being noticed for many years [30], it should be admitted that the actual VL endemic situation might be greatly underestimated in China, which is similar in other endemic countries [31,32]. Poverty is a major determinant for VL endemic regions, most VL patients and threatened communities live in poverty-stricken regions of west China, with poor access to hygiene services, and with extremely poor levels of diagnosis and treatment, which easily leads to misdiagnosis and missed diagnosis for VL, especially for those too weak e.g. infants, to express their feelings. At the same time, results of a previous pilot study showed that the asymptomatic infection rate of VL was fairly high [33,34]. Therefore, mass screening among high risk groups based on symptom monitoring and clue investigation should be conducted to verify whether the current infantile VL cases in China are the tip of the iceberg. In addition, it is necessary to compulsorily register the identity card number of cases during case reporting in NIDRS, which will be of benefit for excluding repeated cases.

It is crucial to know about the current diagnostic approaches for VL employed in China. At present, definitive diagnosis has relied on identification of the parasite by microscopy in organ smears in China. However, it was too difficult to collect and identify parasites in bone marrow for almost all hospitals and CDC laboratories in endemic areas. Of these 1093 infantile VL cases, only 8 cases were detected by etiological diagnosis. As was shown from records of NIDRS, we could merely get limited information of *Leishmania* was detected by microscopy. However, it was still unclear which genus they belonged to. Fortunately, rK39 dipstick was one of the most rapid and easy to interpret diagnostic methods, with a relatively high sensitivity and specificity. Since 2000, many field trial results had proved the rK39 dipstick’s effect on diagnosis of VL in China, comparing with that of pathogenic examination, the concordance rate ranged from 96.07% to 100.00% [35-38]. rK39 dipstick had been extensively employed to detect *Leishmania*-specific antibodies, and played an irreplaceable role in diagnosis of VL and suspected VL cases in China. In addition, immunological methods of enzyme linked immunosorbent assay (ELISA) were used for detection of specific antibodies of VL cases [39,40]. In recent years, molecular method was also established for the detection of *Leishmania* species [41-44]. The samples were tested by PCR using specific pairs of primers, for detecting *Leishmania*-specific DNA. However, the application of molecular techniques was greatly restricted in endemic areas, due to the lack of technicians and equipment.

At present, AVL is endemic in the old oasis areas of west Xinjiang Uygur Autonomous Region, mainly affecting the elder children and adolescents. MST-ZVL is endemic in mountainous areas of southern Gansu Province and northern Sichuan Province, most cases are children less than 10 years old, and the incidence rates for infants are relatively high, ranging from 66.7% to 85.4% [45-47]. DST-ZVL is endemic in certain desert areas of northwest China, and more than 90% cases are infants [48,49]. According to the endemic types of each county, 56.63% (619/1093) cases come from DST-ZVL endemic counties, 37.69% (412/1093) from MST-ZVL endemic counties, and only 5.67% (62/1093) from AVL endemic counties. ZVL is the visibly more serious threat to infants of endemic regions in China, rather than AVL. It is shown that 86.92% (950/1093) of VL cases are infants under 2 years of age, who can not express discomfort and suffering, except crying, which can easily lead to a weakened immune system, and even to death. From the gender perspective, there is no statistically significant difference over time, male and female infants are at the same degree of being threatened by VL. Accordingly, active surveillance and detection should be strengthened for infants in mountainous areas of southern Gansu Province, northern Sichuan Province and in desert areas of west Xinjiang Uygur Autonomous Region, targeting detection, diagnosis and treatment of infantile cases at the early stage so as to protect the health of infants. In China, sodium stibogluconate was recommended and provided as the first-line treatment medicine to patients. Sodium stibogluconate (containing stibonium with 100 mg/ml) was uniquely produced by a government-assigned drug factory. It was reported that the treatment effect was fairly good, and more than 97% patients had been completely cured in two courses of treatment [50,51]. Of course, in the case of relapse, patients will be advised treatment with a second-line medicine, such as amphotericin B [52,53]. It was extensively confirmed that the sodium stibogluconate was relatively safe, and there was no evidence to relate the reported 3 deaths with drug toxicity in this study.

The peak of sandfly density appears from June to September in China [54], which indicates the start of the incidence peak. The temporal clustering and seasonality for infantile VL cases showed most cases occurred in the period of September to February of the next year, which is consistent with results of previous studies [48,49]. The wild *Phlebotomus chinensis* is the major vector of MST-ZVL, and the wild *Phlebotomus wui* and *Phlebotomus alexandri* are the vectors of DST-ZVL [55]. Compared with domestic and semi-domestic sandflies of AVL, vectors of ZVL inhabit the wild, with a large quantity and powerful invasiveness [54]. Unfortunately, people live in poor housing, lack individual protection and consciousness and do not use bed nets in endemic areas in China. In particular, infants are usually directly exposed in courtyards without any protection in summer, when they are at the greatest risk of acquiring sandflies bites and infection. Furthermore, spraying insecticide in ZVL endemic areas showed low effectiveness due to the sandfly’s breeding nature. In the light of current control status, integrated strategies on the control of vector density, health education and scale-up of insecticide-treated bed nets should be considered and trialed.

At the end of the 20th century there were 43 counties where VL prevailed, cases were in all age groups, and AVL was particularly serious at that time. During the study period, 56 counties reported infantile VL cases, which showed that the geographical range of notified infantile VL has significantly expanded in the last decade in China. As shown from the monthly number of infantile VL cases reported, there is a striking variation over time. In general, the endemic situation is stable, except for two peak onsets in certain months. Meanwhile, we also found a high consistency between the monthly number of cases and towns where cases were reported. In other words, as more monthly infantile VL are cases reported, more endemic towns become apparent. It is suggested that if control efforts are not strengthened, the spatial spread of infantile VL will be inevitable in China. There were two main endemic zones identified for infantile VL in China during the study period, indicating the priority areas for investment and targeting.

## Conclusions

VL is a neglected disease and affects the poorest of the poor in western China. The endemic situation of infantile VL is serious, with several active foci identified in China. VL has emerged as a severe threat to infants in endemic regions. Control activities and investments are far from adequate for the current demand. Along with poverty, malnutrition and population growth of non-immunized newborns, infantile VL tends to spread. It is particularly urgent to focus much more attention on this situation and take appropriate actions to control VL in key endemic areas of China.

### Consent

VL was a notifiable infectious disease in China. According to the law, when infants were diagnosed as VL, their detailed information will be provided by parents or guardians, for filling in report cards. The premise was that parents or guardians had learnt and agreed with the items about CDC at different levels were responsible for the collection, analysis and notification of the incidence situation to public. In addition, we did not carry out any field investigation, detection and experiments on patients, it was not necessary to obtain consents from infant’s parents or guardians.

## Competing interests

All authors declare that there are no competing interests in this study.

## Authors’ contributions

QF designed the study and wrote the first version of the manuscript. QF, YYH and SZ implemented the study. SZL, WPW and LHT reviewed the manuscript. SZ, YF and LPZ collected population data of infants and supervised data entry. All authors have read and approved the final manuscript.
